# Supplementary material for: Autophagy sustains glutamate and aspartate synthesis in Saccharomyces cerevisiae during nitrogen starvation
Source: Nat Commun. 2021 Jan 4;12:57. doi: 10.1038/s41467-020-20253-6 (PMC7782722; doi:10.1038/s41467-020-20253-6)
Supplement: Supplementary file 1 — Supplementary Information [file 41467_2020_20253_MOESM1_ESM.pdf]

Autophagy sustains glutamate and aspartate synthesis in *Saccharomyces cerevisiae* during nitrogen starvation

Kuanqing Liu, Benjamin M. Sutter, Benjamin P. Tu

Supplementary Information

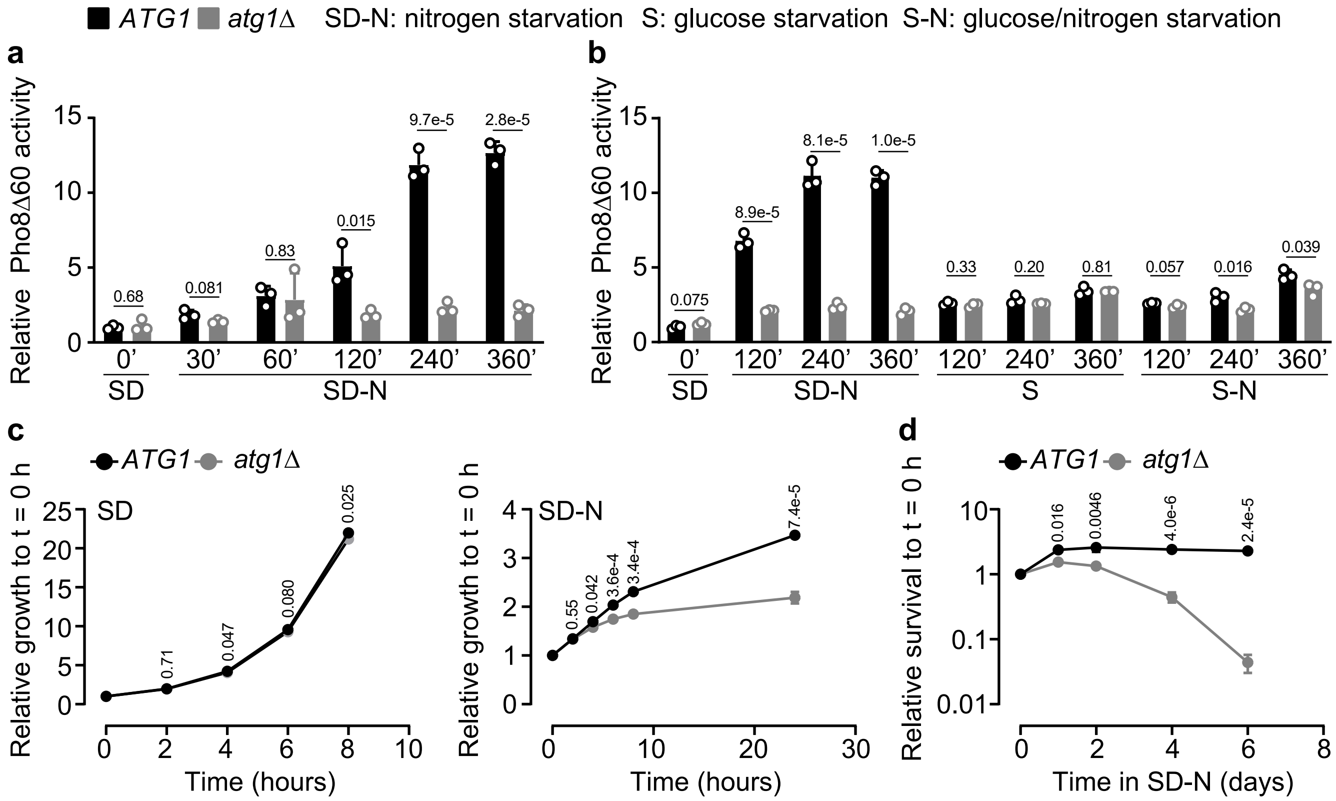

Supplementary Figure 1. **Examination of autophagy induction by various nutrient starvations. a** Nitrogen starvation induces autophagy in WT (*ATG1*) cells, but not in *atg1*Δ cells. Three independent replicates. **b** Starvation for glucose or glucose/nitrogen induces little autophagy in yeast. Nitrogen-starved samples were collected and processed alongside glucose-and glucose/nitrogen-starved samples for direct comparison. Three independent replicates. Both strains express a truncated Pho8p (Pho8Δ60) with *PHO13* deleted. Cells were grown in SD medium to log phase and starved for nitrogen (**a**) or indicated nutrients (**b**) for varying times. Samples were collected and Pho8Δ60 activity was measured as described in Methods. All samples were normalized to WT cells grown in SD (0'). **c** Growth of WT and *atg1*Δ cells in the presence (SD) or absence (SD-N) of nitrogen. Cells were grown in SD to log phase and washed with an equal volume of prewarmed SD or SD-N before being diluted into the corresponding medium with a starting OD_{600} ~0.1. Three independent replicates. **d** Survival of WT and *atg1*Δ cells under prolonged nitrogen starvation. Three independent replicates. P values were calculated using unpaired two-sided Student's *t* test assuming equal variances. Data are presented as mean ± standard deviation.

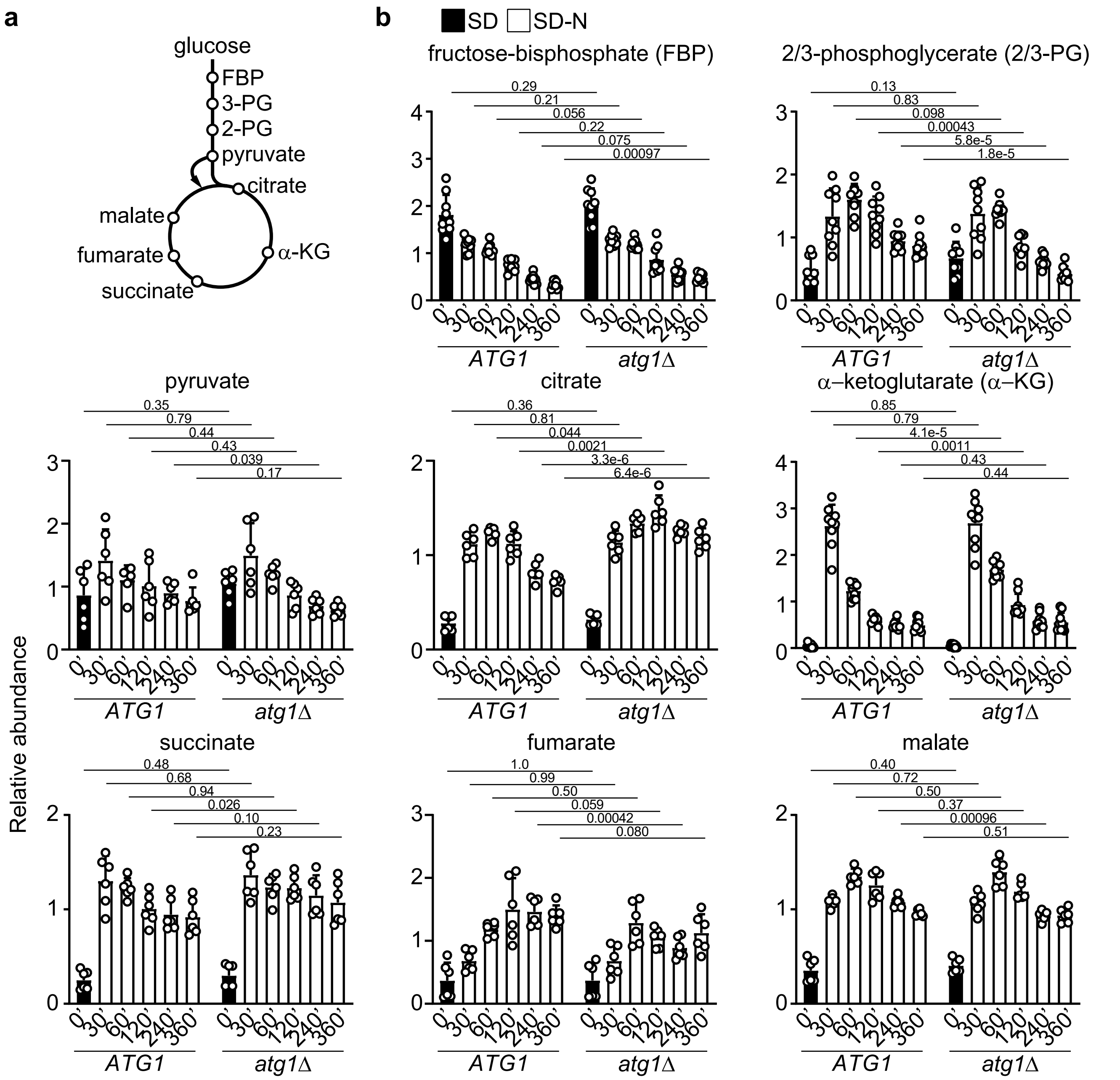

Supplementary Figure 2. **Examination of levels of glycolytic and TCA intermediates in the presence and absence of nitrogen. a** Schematic of simplified glycolysis and TCA in *S. cerevisiae*. Only relevant intermediates are shown. **b** Changes of glycolytic and TCA intermediates in WT (*ATG1*) and *atg1*Δ cells under nitrogen starvation. Nine independent replicates for FBP, 2/3-PG, and α-KG, and six for the remaining metabolites. P values were calculated using unpaired two-sided Student's *t* test assuming equal variances. Data are presented as mean ± standard deviation.

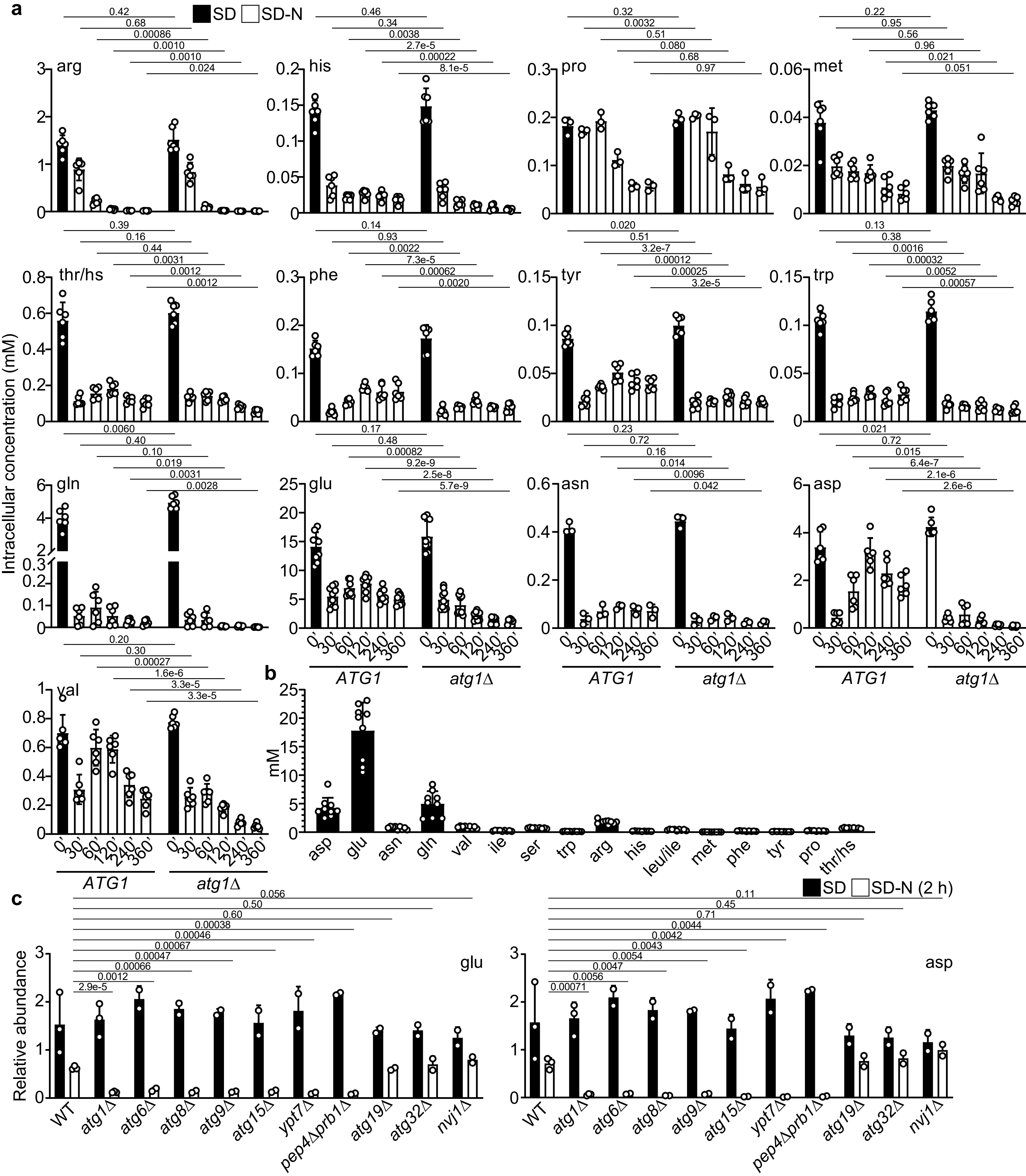

Supplementary Figure 3. **Autophagy is critical for maintaining amino acid pools under nitrogen starvation**. **a** Changes of amino acids in WT (*ATG1*) and *atg1*Δ cells under nitrogen starvation. Samples were collected as depicted in Figure 1a. Shown here are amino acids detected and quantified using a second MRM (multiple reaction monitoring) transition (if available). hs: homoserine. Three independent replicates for pro and asn, nine for glu, and six for the remaining amino acids. **b** Estimation of intracellular amino acid concentration of WT cells grown in SD. Nine independent replicates. **c** Amassing glutamate and aspartate under nitrogen starvation requires an intact autophagy machinery, but not Cvt pathway (*ATG 19*), mitophagy (*ATG32*), or piecemeal microautophagy (*NVJ1*). Three independent replicates for WT and *atg1*Δ, two for the remaining strains. P values were calculated using unpaired two-sided Student's *t* test assuming equal variances. Data are presented as mean ± standard deviation.

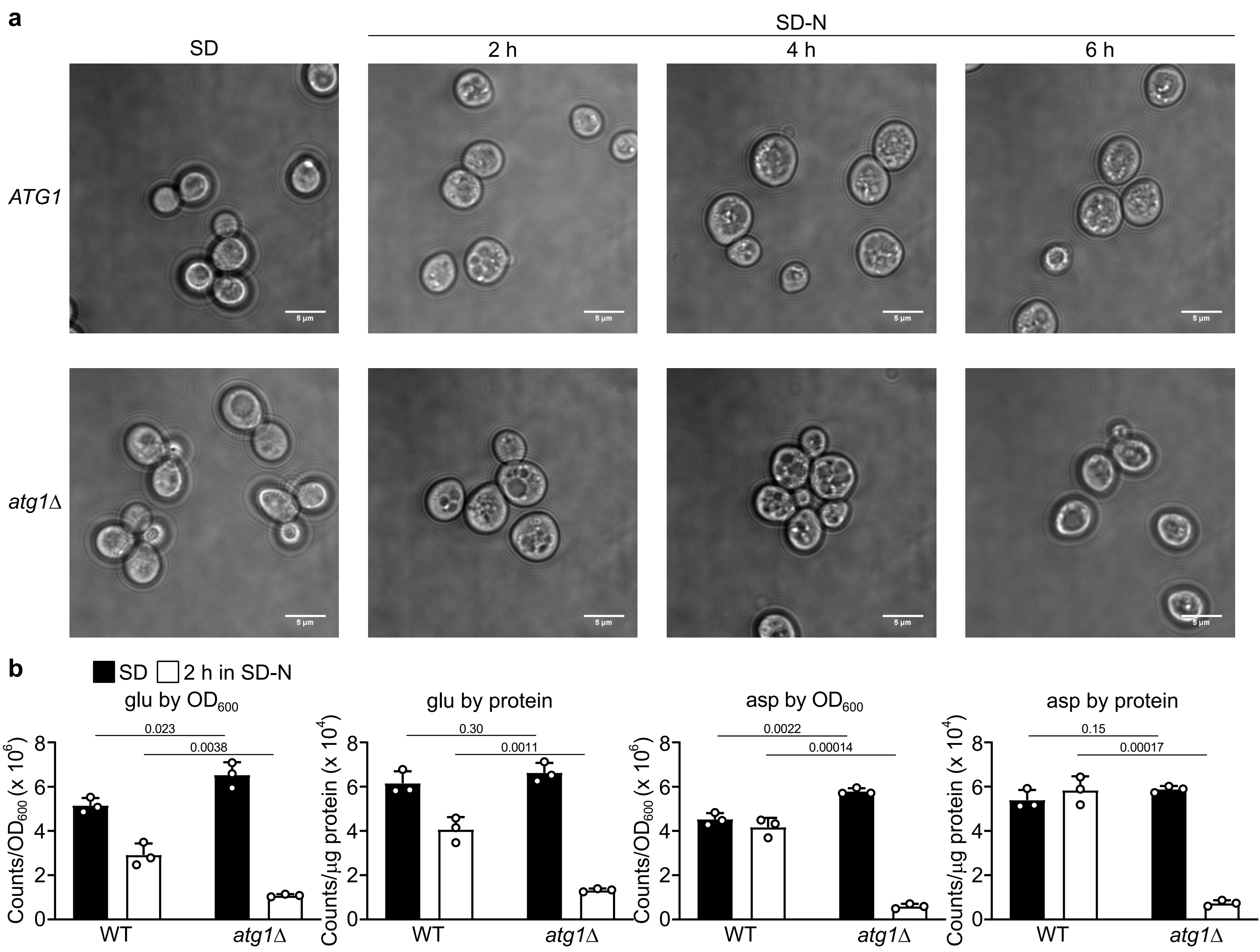

Supplementary Figure 4. **Controls for metabolite data processing**. **a** Examination of cell size in the presence and absence of nitrogen. All the images were taken with identical microscope settings and therefore are directly comparable. This experiment was performed once and multiple images were taken for each strain at each time point. A representative image is shown here and similar results were obtained from additional images (see Source Data). Scale bar: 5 μm. **b** Normalization using OD_{600} and protein gives similar results. Three independent replicates. P values were calculated using unpaired two-sided Student's *t* test assuming equal variances. Data are presented as mean ± standard deviation. Data were also used for plotting Figure 1b.

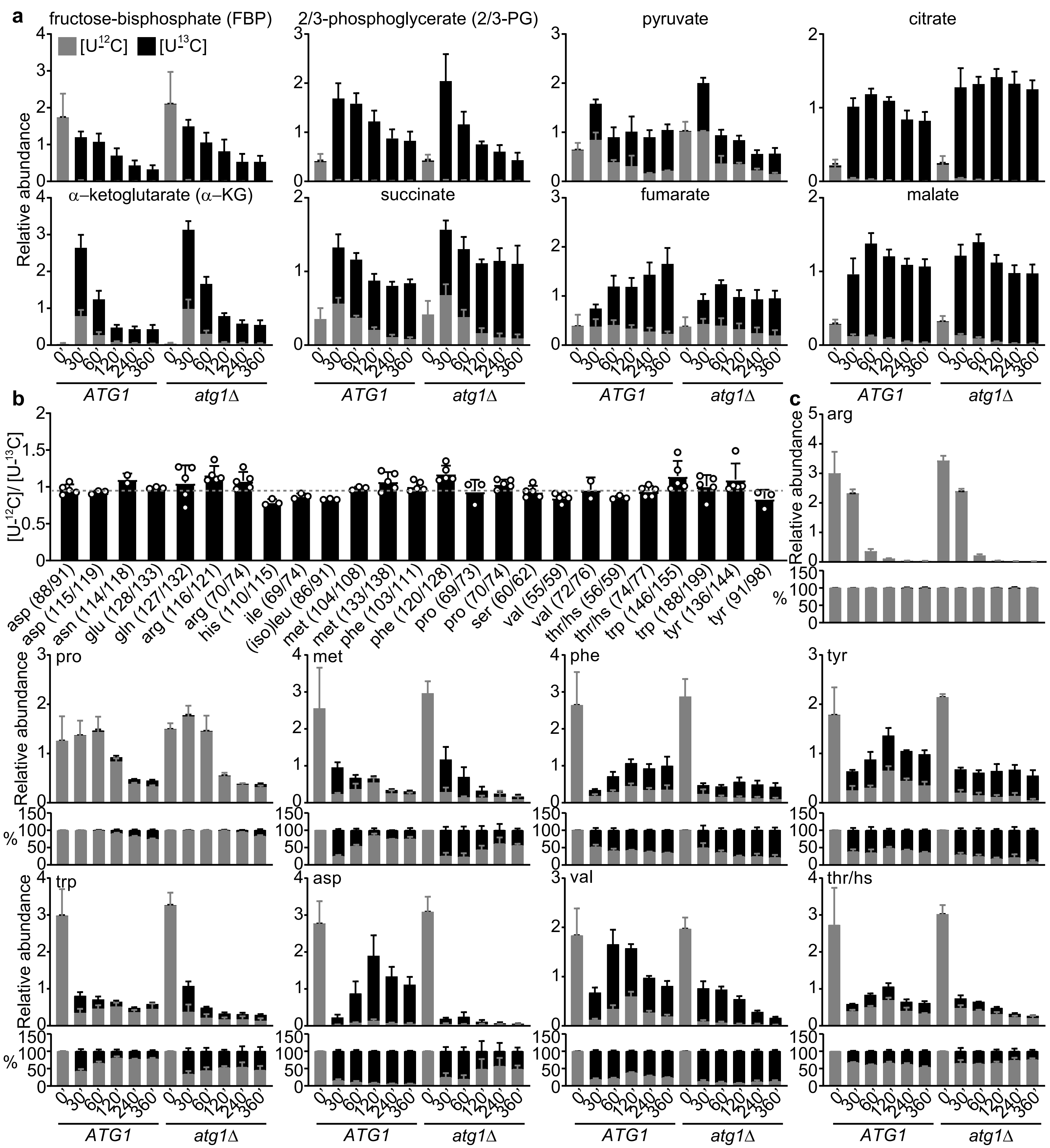

Supplementary Figure 5. **Autophagy promotes the accumulation of specific [U-^{13}C]-amino acids under nitrogen starvation.**
a Changes of [U-^{12}C]- and [U-^{13}C]-glycolytic and TCA intermediates in WT (*ATG1*) and *atg1*Δ cells under nitrogen starvation. Three independent replicates for pyruvate, five for citrate, fumarate, and malate, and eight for the remaining metabolites. **b** [U-^{12}C]-and [U-^{13}C]-amino acids are detected with comparable sensitivities. WT cells were fully labeled in [U-^{12}C]-or [U-^{13}C] by growing cells in the corresponding SD medium for ~12 doublings. Equal OD$_{600}$ of cells were mixed and metabolites were extracted and measured. Numbers in parentheses correspond to the [U-^{12}C]- and [U-^{13}C]-daughter ions of an amino acid, respectively. Two technical replicates for asn_114/118, val_72/76, three for asp_115/119, glu_128/133, his_110/115, ile_69/74, ile/leu_86_91, met_104/108, pro_69/73, thr/hs_56/59, and tyr_91/98, and five for the remaining transitions. **c** Changes of [U-^{12}C]- and [U-^{13}C]-amino acids in WT and *atg1*Δ cells under nitrogen starvation. Shown here are amino acids detected using their second MRM transition (if available). hs: homoserine. Six independent replicates for phe, trp, asp, and val, and three for the remaining amino acids. Data are presented as mean ± standard deviation.

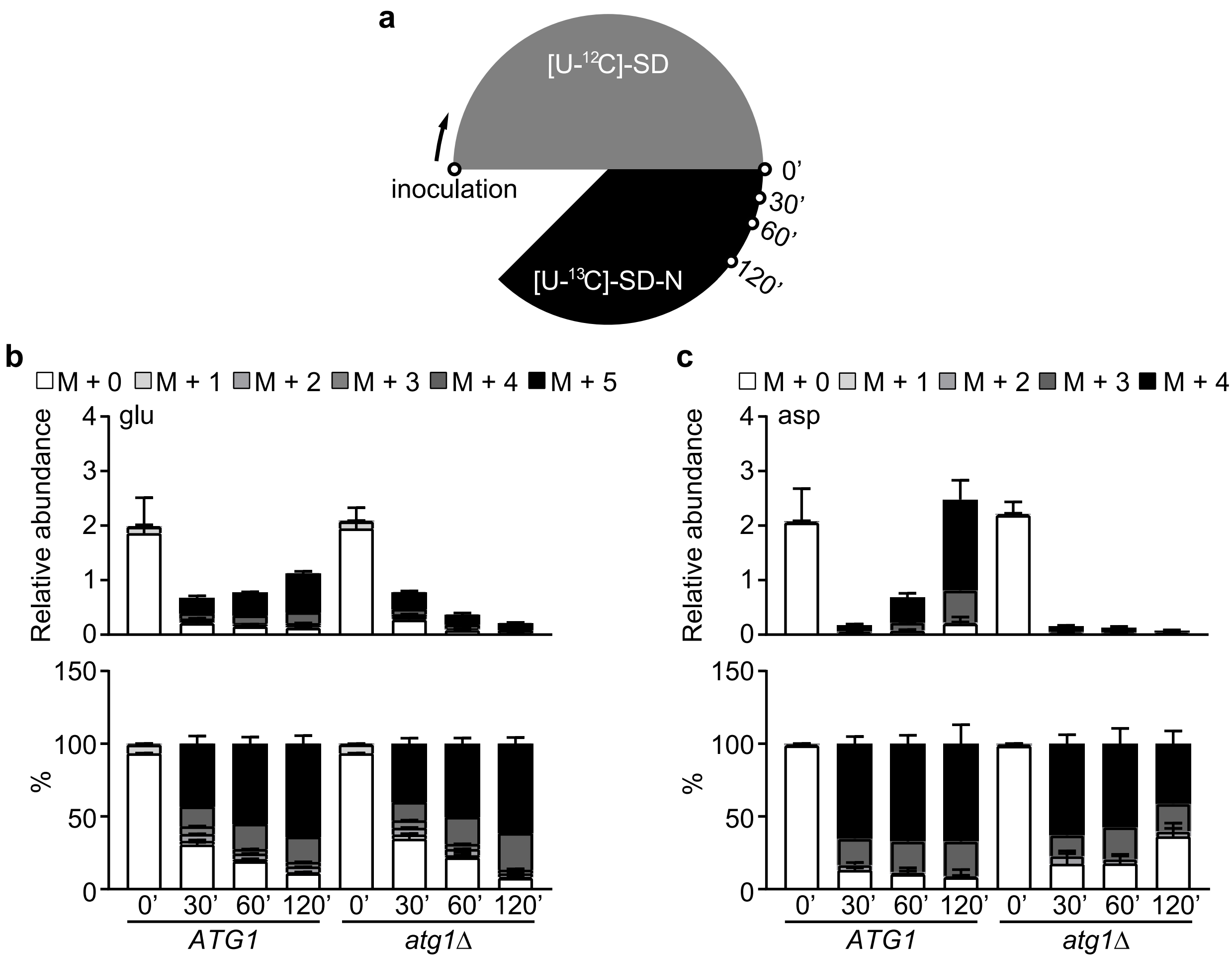

Supplementary Figure 6. **Assimilation of glucose-derived ^{13}C into isotopologues of glutamate and aspartate in WT (*ATG1*) and *atg1*Δ cells under nitrogen starvation. a** Schematic of the experimental design. **b** ^{13}C-isotopologues of glutamate under nitrogen starvation. Three independent replicates. **c** ^{13}C-isotopologues of aspartate under nitrogen starvation. Three independent replicates. The top panel shows the relative abundance of each isotopologue, whereas the bottom graph represents the fraction of each isotopologue. Data are presented as mean ± standard deviation. Data from **b** and **c** were also used to plot Figure 2b.

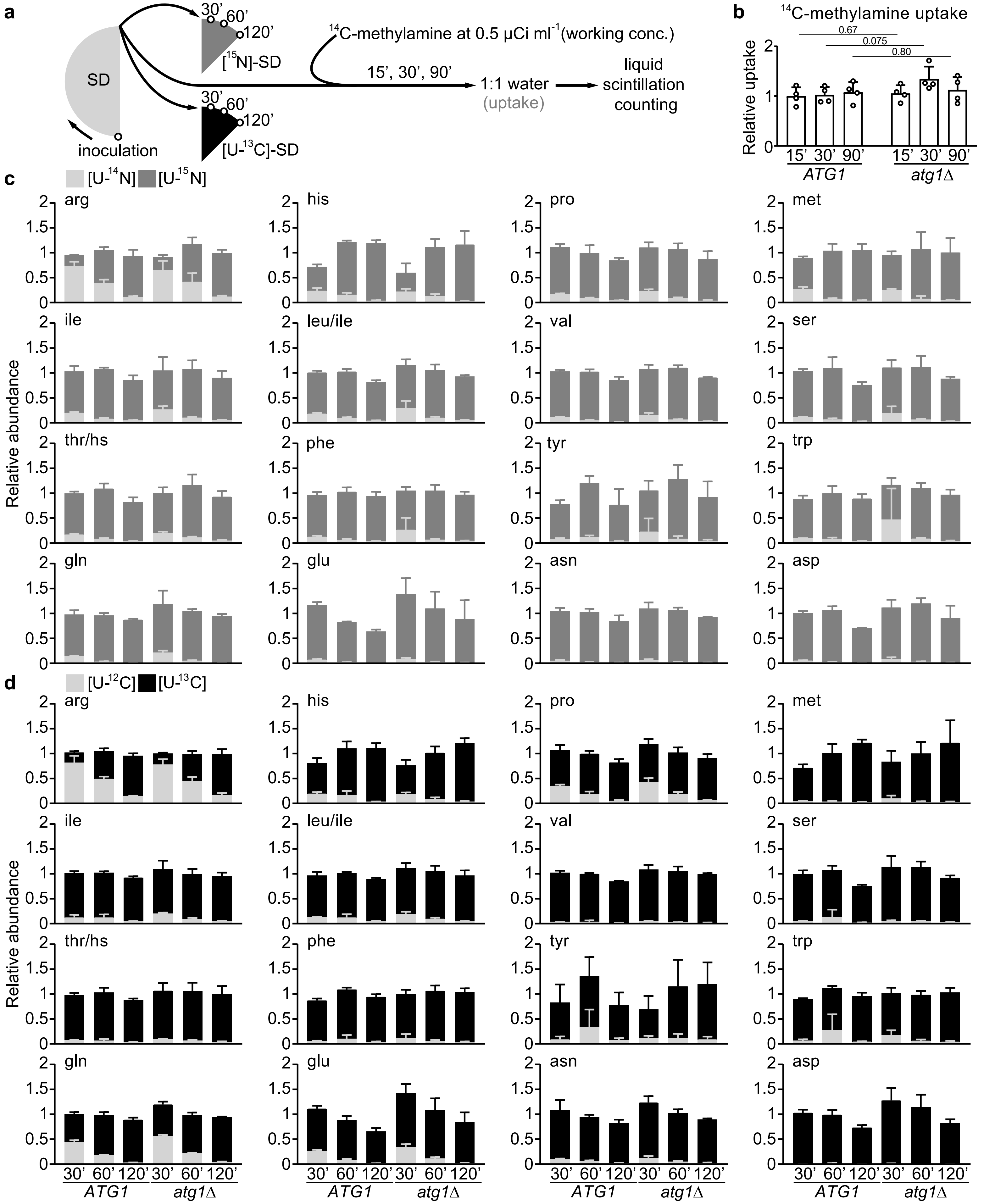

Supplementary Figure 7. **Assimilation of ammonium and glucose into amino acids is similar between WT (*ATG1*) and *atg1Δ* cells in SD.** **a** Schematic of the experimental design for **b**, **c**, and **d**. **b** Uptake of the ammonium analog methylamine. CPM (counts per minute) was normalized to OD_{600} and then to WT at 15'. Four independent replicates. **c** Incorporation of ammonium into amino acids in WT and *atg1Δ* cells in SD. Three independent replicates. **d** Incorporation of glucose-derived carbon into amino acids in WT and *atg1Δ* cells in SD. Three independent replicates. hs: homoserine. P values were calculated using unpaired two-sided Student's *t* test assuming equal variances. Data are presented as mean ± standard deviation.

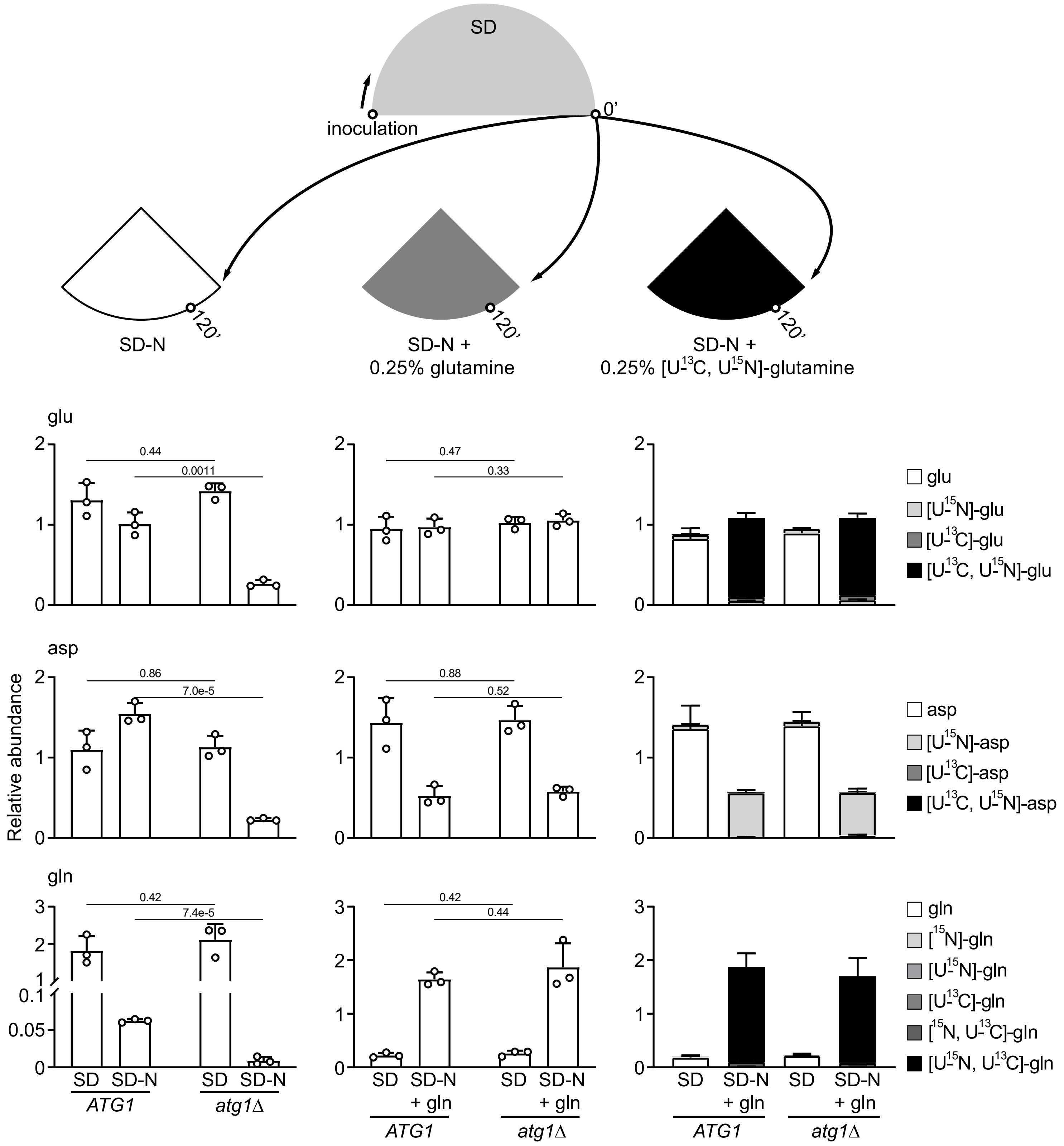

Supplementary Figure 8. **Glutamine rescues glutamate and aspartate levels in *atg1*Δ cells under nitrogen starvation.** Three independent replicates. P values were calculated using unpaired two-sided Student's *t* test assuming equal variances. Data are presented as mean ± standard deviation.

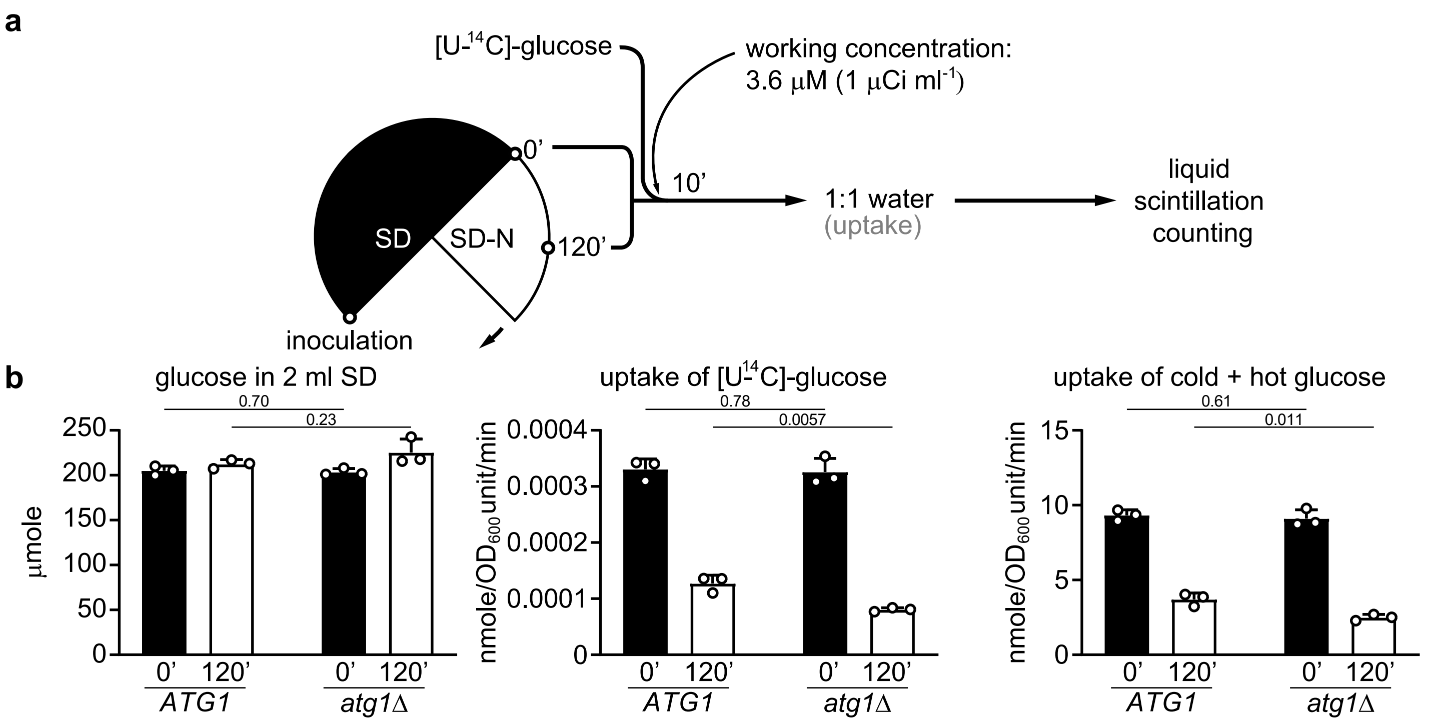

Supplementary Figure 9. **Examination of glucose uptake in WT (*ATG1*) and *atg1*Δ cells**. **a** Schematic of the experimental design for measuring glucose uptake. Two-ml log-phase culture was labeled with [U-^{14}C]-glucose for 10 minutes and mixed with an equal volume of ice-cold water. Cells were loaded onto a glassfiber filter (Whatman GF/F, GE Healthcare) and washed with ice-cold water. Filters were dried and counted in Ultima Gold F (Perkin Elmer) using a Hitachi liquid scintillation counter (AccuFlex LSC-8000). **b** Autophagy deficiency slightly decreases glucose uptake under nitrogen starvation. Extracellular glucose was measured using a glucose assay kit (Sigma) following the manufacturer's instructions. Three independent replicates. P values were calculated using unpaired two-sided Student's *t* test assuming equal variances. Data are presented as mean ± standard deviation.

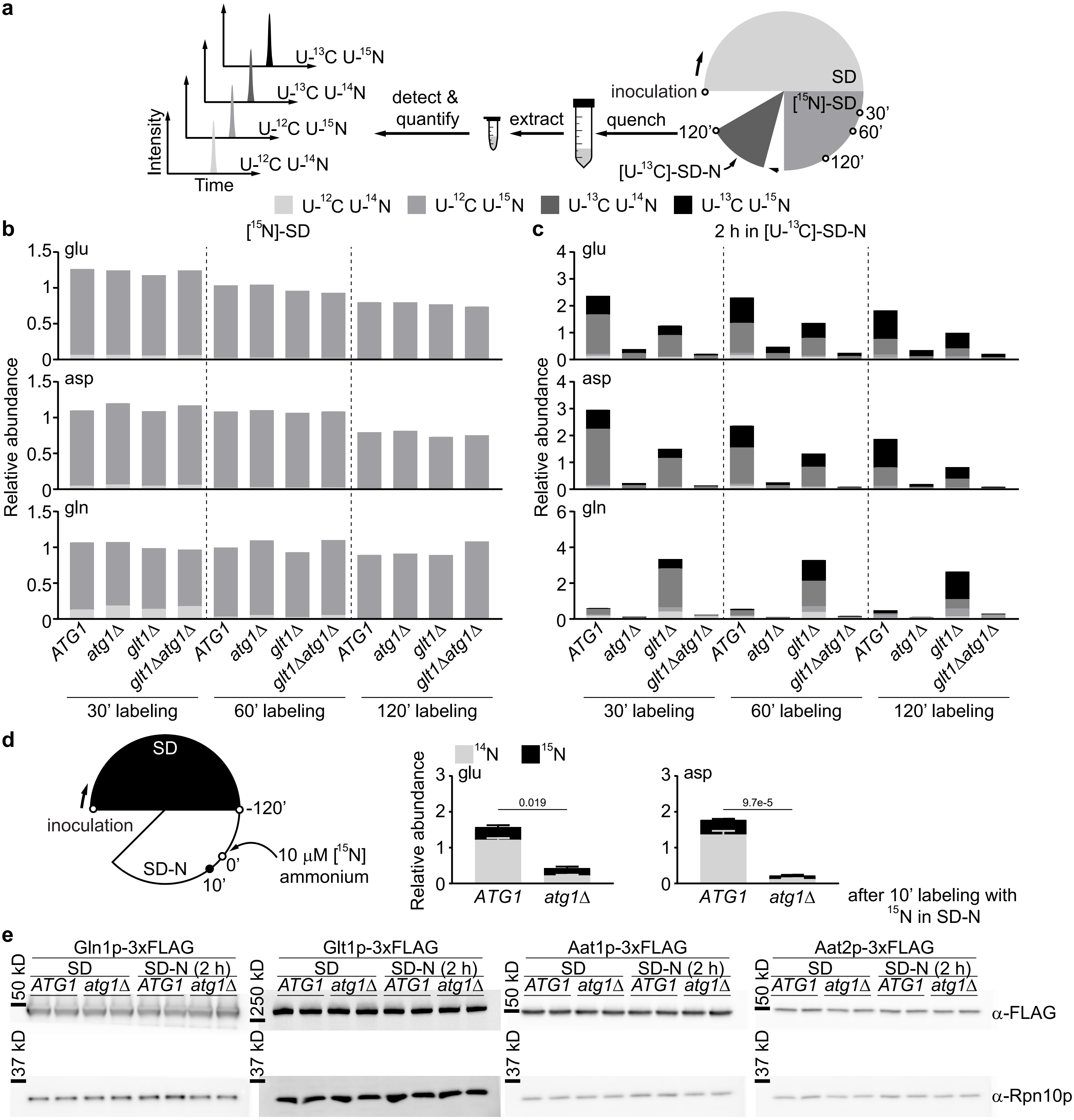

Supplementary Figure 10. **Autophagy enables yeast cells to retrieve ammonium to support glutamate and aspartate synthesis under nitrogen starvation. a** Schematic of the experimental design for **b** and **c**. Yeast cells were grown in SD medium to log phase and switched to [^{15}N-SD] for varying times before being shifted to [U-^{13}C]-SD-N for two hours. Metabolites were collected and analyzed by LC-MS/MS. **b** Assimilation of ^{15}N into glutamate, aspartate, and glutamine is comparable among various yeast strains in SD. Two independent replicates. **c** Autophagy enables yeast cells to retrieve ammonium to support synthesis of glutamine, glutamate, and aspartate under nitrogen starvation. Shown here are relative levels of differentially labeled metabolites after two-hour nitrogen starvation. Two independent replicates. **d** Assimilation of exogenous ammonium into glutamate and aspartate under nitrogen starvation. Three independent replicates. Only ^{15}N-glutamate and -aspartate were compared for statistical significance. Nitrogen-starved cells were fed with 5 μM $(^{15}NH_4)_2SO_4$. **e** Protein levels of Gln1p, Glt1p, Aat1p, and Aat2p do not change significantly between WT (*ATG1*) and *atg1*Δ cells before and after nitrogen starvation. This experiment was performed twice with similar results. Shown here are both replicates, with approximate molecular size markers indicated (black line). See Source Data for uncropped images. P values were calculated using unpaired two-sided Student's *t* test assuming equal variances. Data are presented as mean only for **b** and **c**, and mean ± standard deviation for **d**.

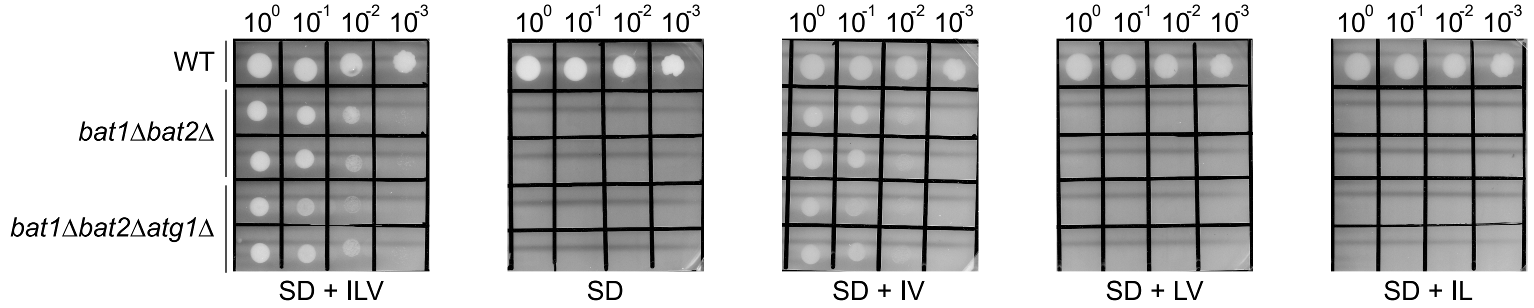

Supplementary Figure 11. **Loss of Bat1p and Bat2p leads to auxotrophy for valine and isoleucine, but not leucine.** Cells were grown in SD + isoleucine, leucine, and valine (ILV) at 1 mM each to log phase. Cells were spun down, washed in sterile water and resuspended in sterile water to a final OD_{600}~1. Serially diluted samples were spotted onto appropriate agar plates. WT was examined once, whereas *bat1*Δ*bat2*Δ and *bat1*Δ*bat2*Δ*atg1*Δ were tested twice with similar results. Shown here are both replicates. See source data for the uncropped images.

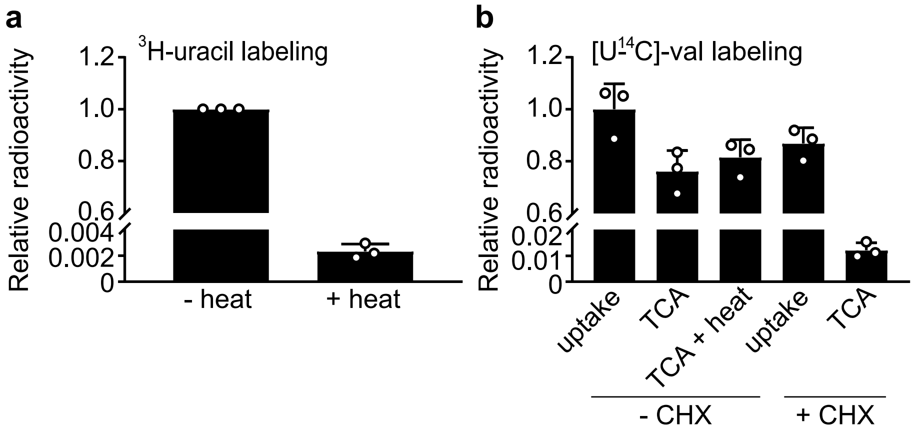

Supplementary Figure 12. **Controls for radioisotope tracing experiments.**
a Heat treatment in 10% TCA effectively solubilizes precipitated nucleic acids. WT cells were grown in SD to log phase and [5,6-^{3}H]-uracil (Perkin Elmer) was added to a final concentration of 1 μCi ml^{-1}. Cells were labeled for 10 minutes and two aliquots of cell culture were mixed with an equal volume of ice-cold TCA (final concentration at 10%). Samples were incubated on ice for 10 minutes. One sample was heated at ~95℃ for 10 minutes and chilled on ice. Samples were then transferred to a Whatman GF/F filter (GE Healthcare), washed with 5% ice-cold TCA, and dried overnight. Filters were counted in Ultima Gold F (Perkin Elmer) using a Hitachi liquid scintillation counter (AccuFlex LSC-8000). Radioactivity was normalized to unheated samples. Three independent replicates.
b Heat treatment does not solubilize TCA-precipitated proteins. [U-^{14}C]-valine (Perkin Elmer) labeling experiment was performed similarly as described above and it was added to a final concentration of 0.271 μCi ml^{-1}(equivalent to 1 μM valine). Cycloheximide (CHX) (final concentration at 25 μg ml^{-1}) was added simultaneously with [U-^{14}C]-valine to inhibit translation. Samples for uptake were mixed with an equal volume of ice-cold water and washed with ice-cold water. Radioactivity was normalized to uptake samples without CHX. Three independent replicates. Data are presented as mean ± standard deviation.

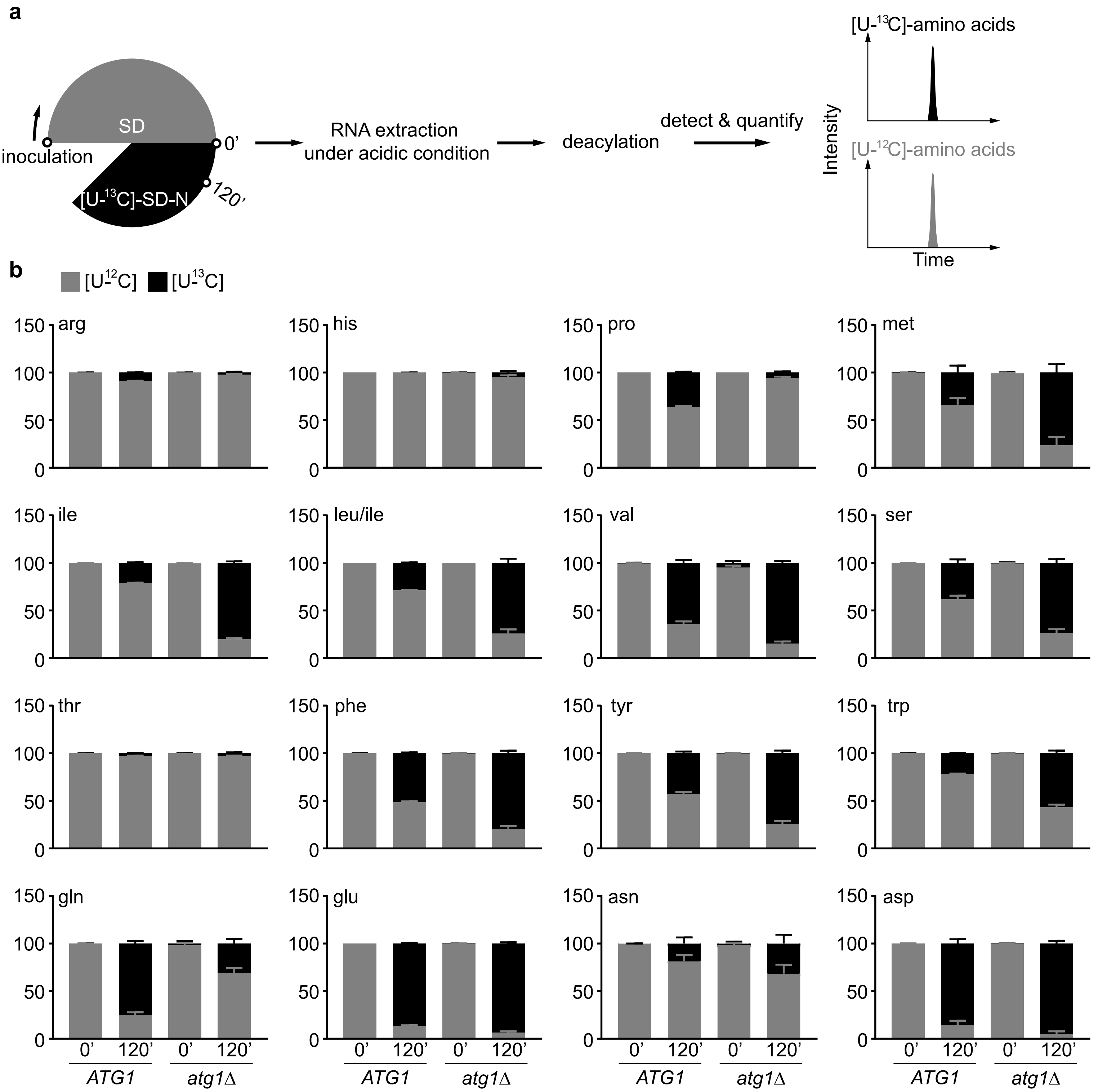

Supplementary Figure 13. **Fraction of [U-^{12}C]- and [U-^{13}C]-amino acids aminoacylated onto tRNAs.**
a Schematic of the experimental design. See Methods for details. **b** Fractions of [U-^{12}C]- and [U-^{13}C]-amino acids aminoacylated onto tRNA. Three independent replicates. Data are presented as mean ± standard deviation.

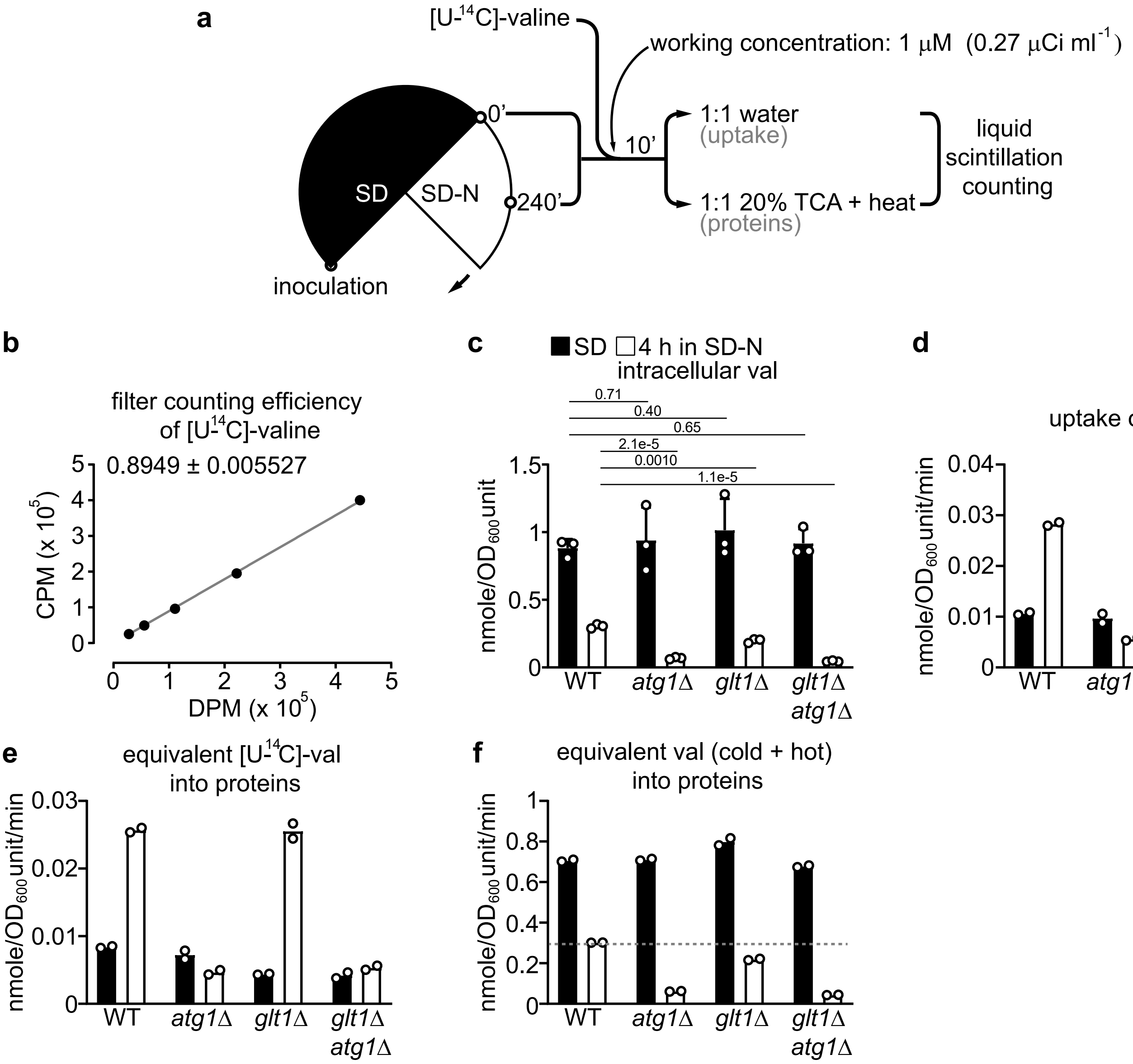

Supplementary Figure 14. **The GS-GOGAT pathway supports translation under nitrogen starvation.** **a** Schematic of the experimental design. **b** Counting efficiency of [U-^{14}C]-valine on filters. An aliquot of 40 μl of serially diluted [U-^{14}C]-valine (0.2, 0.1, 0.05, 0.025, and 0.0125 μCi) was spotted evenly onto a glass fiber filter (Whatman GF/F, GE Healthcare). Filters were dried and counted in Ultima Gold F (Perkin Elmer) using a Hitachi liquid scintillation counter (AccuFlex LSC-8000). One replicate. **c** Estimation of intracellular valine. Three independent replicates. **d** Uptake of [U-^{14}C]-valine. Two independent replicates. **e** Incorporation of equivalents of [U-^{14}C]-valine (valine and its derivatives) into proteins. Two independent replicates. **f** Incorporation of total valine equivalents (radioactive/hot and non-radioactive/cold) into proteins. Two independent replicates. P values were calculated using unpaired two-sided Student's *t* test assuming equal variances. Data are presented as mean ± standard deviation for **c** and as mean only for **d**-**f**.

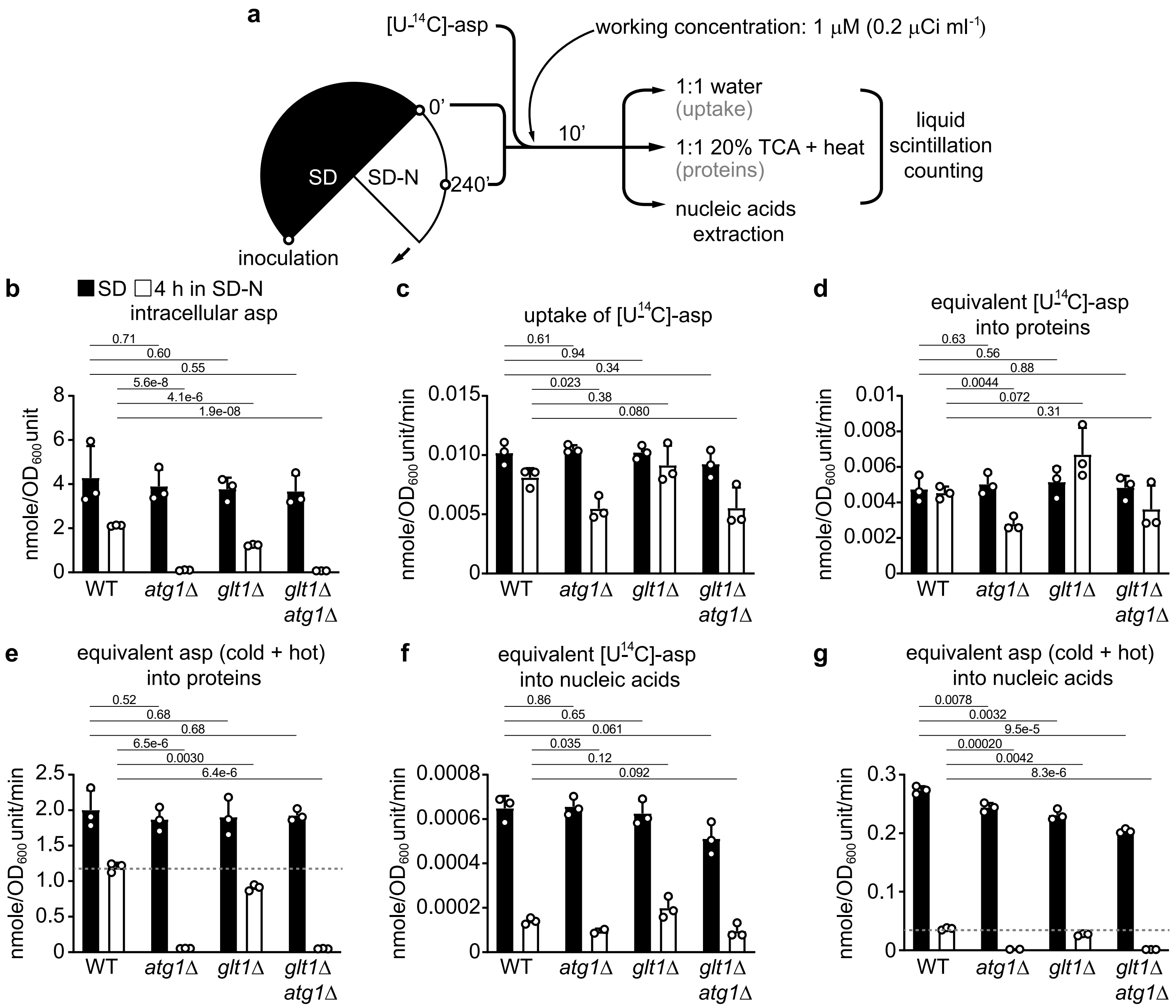

Supplementary Figure 15. **The GS-GOGAT pathway supports macromolecule synthesis under nitrogen starvation.**
a Schematic of the experimental design. **b** Estimation of intracellular aspartate. Three independent replicates. **c** Uptake of [U-^{14}C]-aspartate. Three independent replicates. **d** Incorporation of equivalents of [U-^{14}C]-aspartate (aspartate and its derivatives) into proteins. Three independent replicates. **e** Incorporation of total aspartate equivalents (radioactive/hot and non-radioactive/cold) into proteins. Three independent replicates. **f** Incorporation of equivalents of [U-^{14}C]-aspartate into nucleic acids. Two independent replicates for *atg1*Δ in SD-N for 4 h, and three for the remaining strains. **g** Incorporation of total aspartate equivalents into nucleic acids. Two independent replicates for *atg1*Δ in SD-N for 4 h, and three for the remaining strains. P values were calculated using unpaired two-sided Student's *t* test assuming equal variances. Data are presented as mean ± standard deviation.

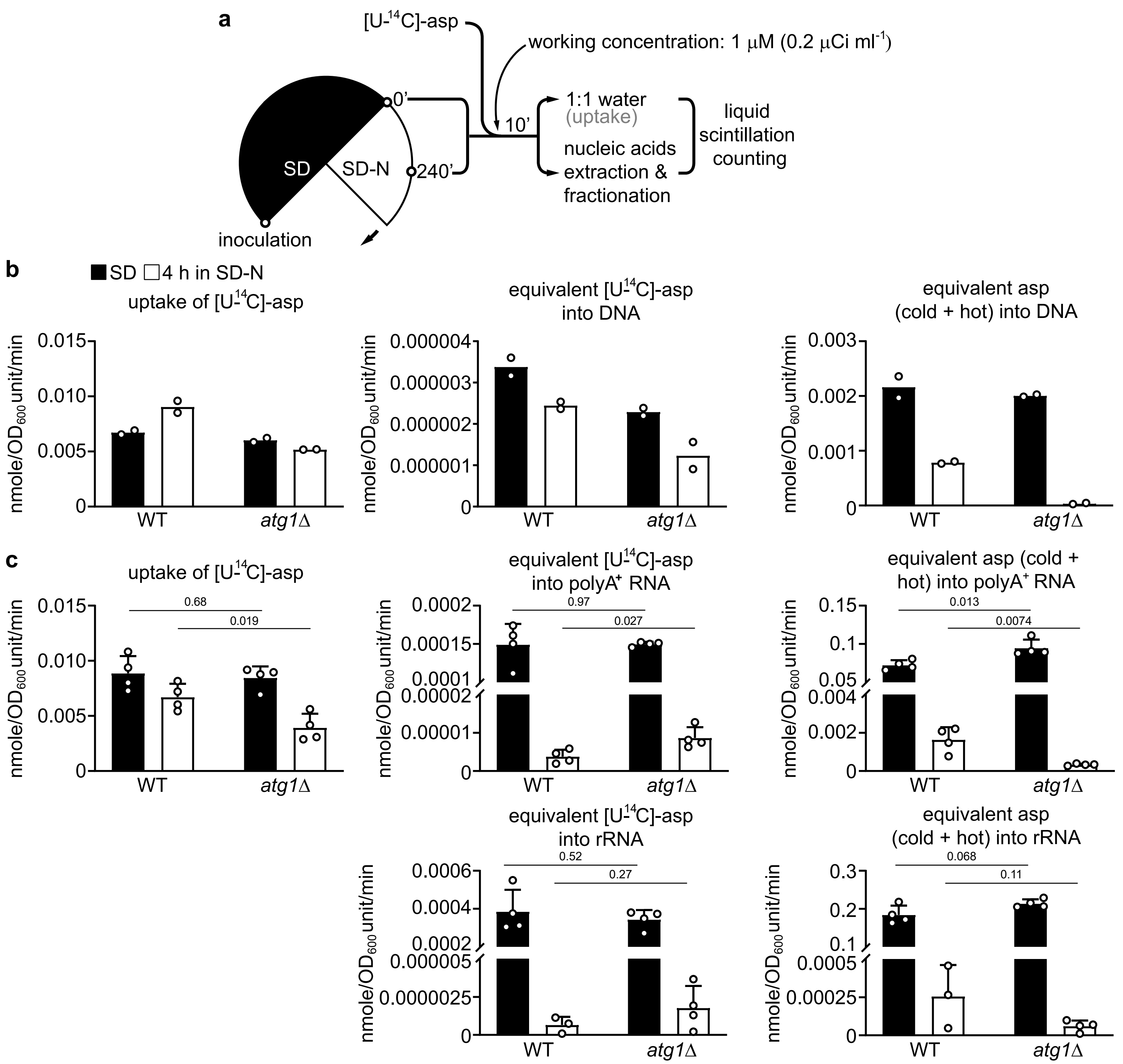

Supplementary Figure 16. **Tracing of aspartate into different classes of nucleic acid in the presence and absence of nitrogen.** **a** Schematic of the experimental design. **b** Incorporation of equivalents of aspartate (aspartate and its derivatives) into DNA. Two independent replicates. **c** Incorporation of equivalents of aspartate into polyA$^+$ RNA and rRNA (18S and 25S rRNA). Three independent replicates for rRNA of WT in SD-N for 4 h and four independent replicates for the remaining strains. Aspartate concentration from Figure 7c was used to calculate the incorporation of total aspartate equivalents into different classes of nucleic acid. P values were calculated using unpaired two-sided Student's *t* test assuming equal variances. Data are presented as mean only for **b** and mean ± standard deviation for **c**.

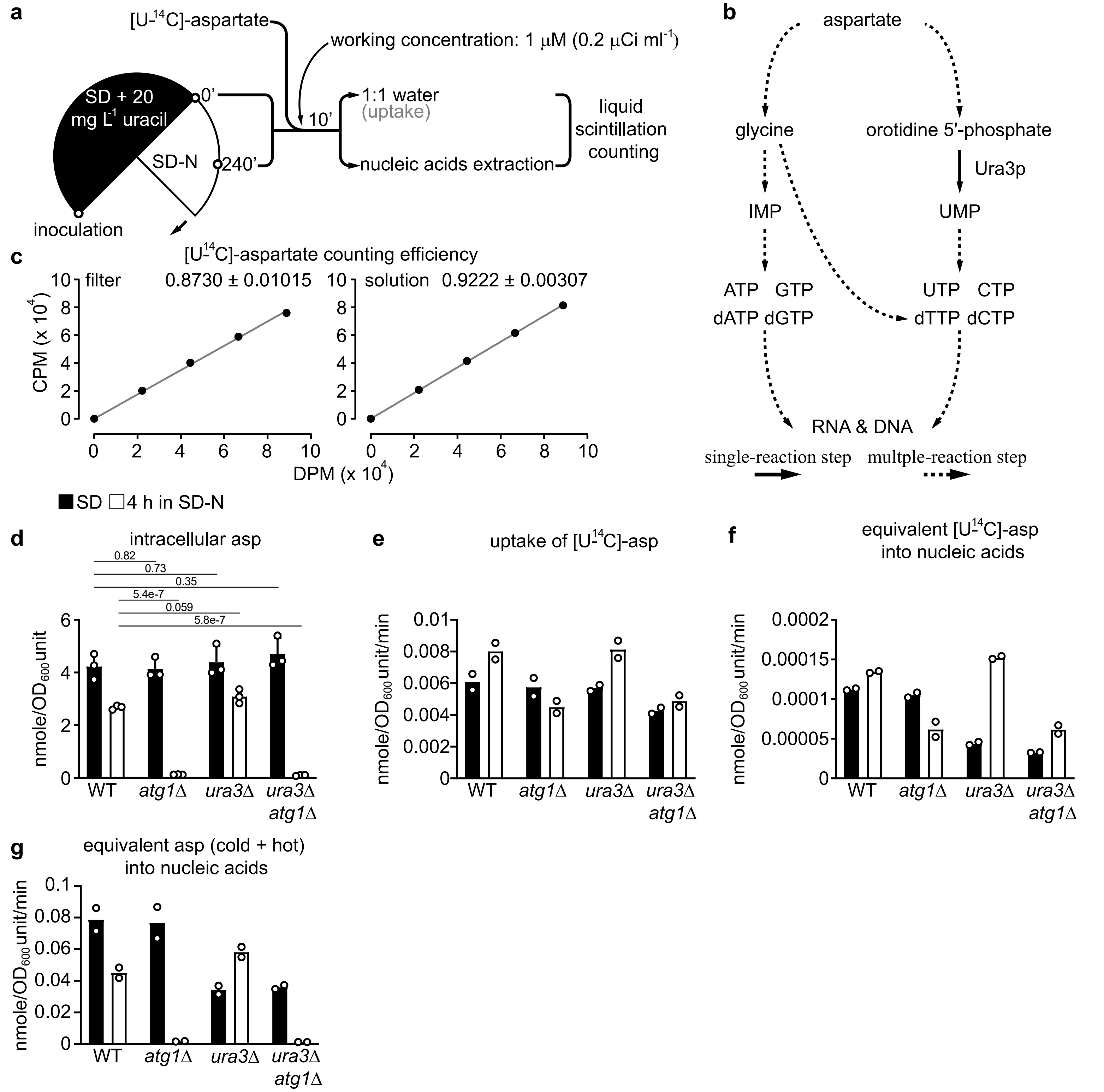

Supplementary Figure 17. **Impact of inactivating *de novo* pyrimidine synthesis on aspartate assimilation into nucleic acids.** **a** Schematic of the experimental design. **b** Potential routes for assimilating aspartate-derived carbon into nucleic acids. IMP: inosine monophosphate; UMP: uridine monophosphate. **c** Counting efficiency of [U-^{14}C]-aspartate on filters or in solution. An aliquot of 40 μl of serially diluted [U-^{14}C]-aspartate (0.01, 0.02, 0.03, and 0.04 μCi) was spotted evenly onto a glass fiber filter (Whatman GF/F, GE Healthcare) or directly into Ultima Gold (Perkin Elmer) and counted using a Hitachi liquid scintillation counter (AccuFlex LSC-8000). Filter samples were dried and counted in Ultima Gold F (Perkin Elmer). One replicate. **d** Estimation of intracellular aspartate concentration. Three independent replicates. **e** Uptake of [U-^{14}C]-aspartate. Two independent replicates. **f** Incorporation of equivalents of [U-^{14}C]-aspartate (aspartate and its derivatives) into nucleic acids. Two independent replicates. **g** Incorporation of total aspartate equivalents (radioactive/hot and non-radioactive/cold) into nucleic acids. Two independent replicates. P values were calculated using unpaired two-sided Student's *t* test assuming equal variances. Data are presented as mean ± standard deviation for **d** and mean only for **e-g**.

Supplementary Table 1. Strains, plasmids, antibodies, and isotopes

	**Strains**	
**Name**	**Genotype**	**References**
ATG1	*MATalpha* CEN.PK prototroph	[1]
ATG1/ATG1	*MATa/alpha* CEN.PK prototroph	[1]
atg1Δ	*MATalpha atg1::hphMX6*	This work
atg6Δ	*MATalpha atg6::hphMX6*	This work
atg8Δ	*MATalpha atg8::hphMX6*	This work
atg15Δ	*MATalpha atg15::hphMX6*	This work
ypt7Δ	*MATalpha ypt7::hphMX6*	This work
pep4Δ prb1Δ	*MATalpha pep4::hphMX6 prb1:: kanMX6*	This work
atg19Δ	*MATalpha atg19::hphMX6*	This work
atg32Δ	*MATalpha atg32::hphMX6*	This work
nvj1Δ	*MATalpha nvj1::hphMX6*	This work
glt1Δ	*MATalpha glt1::hphMX6*	This work
glt1Δ atg1Δ	*MATalpha glt1::hphMX6 atg1::kanMX6*	This work
gdh1Δ gdh3Δ glt1Δ	*MATalpha gdh1::hphMX6 gdh3::kanMX6 glt1::natMX6*	This work
gdh1Δ gdh3Δ glt1Δ atg1Δ	*MATalpha gdh1::hphMX6 gdh3::kanMX6 glt1::natMX6 atg1::Sh ble*	This work
pho8Δ60 pho13Δ	*MATa pho8::*P_{TEF1}*-pho8Δ60-kanMX6 pho13::natMX6*	[2]
pho8Δ60 pho13Δ atg1Δ	*MATa pho8::*P_{TEF1}*-pho8Δ60-kanMX6 pho13::natMX6 atg1::hphMX6*	This work
aat1Δ	*MATalpha aat1::hygMX6*	This work
aat1Δ atg1Δ	*MATalpha aat1::hygMX6 atg1::natMX6*	This work
aat2Δ	*MATalpha aat2::kanMX6*	This work
aat2Δ atg1Δ	*MATalpha aat2::kanMX6 atg1::natMX6*	This work
aat1Δ aat2Δ	*MATalpha aat1::hygMX6 aat2::kanMX6*	This work
aat1Δ aat2Δ atg1Δ	*MATalpha aat1::hygMX6 aat2::kanMX atg1::nat*	This work
aro8Δ aro9Δ	*MATalpha aro8::natMX6 aro9::Sh ble*	This work
aro8Δ aro9Δ atg1Δ	*MATalpha aro8::natMX6 aro9::Sh ble atg1::hphMX6*	This work
bat1Δbat2Δ	*MATalpha bat1::hphMX6 bat2::KanMX*	This work
bat1Δbat2Δatg1Δ	*MATalpha bat1::hphMX6 bat2::KanMX atg1::nat*	This work
ura3Δ	*MATalpha ura3::KanMX*	This work
ura3Δatg1Δ	*MATalpha ura3::KanMX atg1::hphMX6*	This work
	**Plasmids**	
**Name**	**Description**	**References**
pFA6a-*hphMX6*	for replacing a gene of interest with the *hphMX6* gene	[3]
pFA6a-*kanMX6*	for replacing a gene of interest with the *kanMX6* gene	[3]
pFA6a-*natMX6*	for replacing a gene of interest with the *natMX6* gene	[3]
pUG66	for replacing a gene of interest with the *Sh ble* gene	[4]
	**Antibodies**	
**Name**	**Description**	**Cat. #**
α-FLAG	To detect the FLAG epitope	Sigma F1804/Cell Signaling #2368s
α-Rpn10	To detect Rpn10p	Abcam ab98843
	**Isotopes**	
**Name**	**Vendor**	**Cat. #**

[U-^{14}C]-aspartate	Perkin Elmer	NEC268E050UC
[U-^{14}C]-valine	Perkin Elmer	NEC291EU050UC
[U-^{14}C]-glucose	Perkin Elmer	NEC042X050UC
[5,6-^{3}H]-uracil	Perkin Elmer	NET368250UC
^{14}C-methylamine	American Radiolabeled Chemicals	ARC0167
[U-^{13}C]-glucose	Sigma	389374
[U-^{13}C]-glucose	Cambridge Isotope Laboratories	CLM-1396
^{15}N-ammonium sulfate	Cambridge Isotope Laboratories	NLM-713
[U-^{15}N, U-^{13}C]-glutamine	Cambridge Isotope Laboratories	CNLM-1275-H

Supplementary Table 2. MRM transitions for AB SCIEX 3200

Metabolites	Parental mass	[U-^{12}C] Q_1	[U-^{12}C] Q_3	[U-^{15}N] Q_1	[U-^{15}N] Q_3	[U-^{13}C] Q_1	[U-^{13}C] Q_3	Methods
met	149	150	133<br>104	151	105	155	138<br>108	formic acid
pro	115	116	70<br>69	117	71	121	74<br>73	formic acid
arg	174	175	70<br>116	179	117	181	74<br>121	formic acid
val	117	118	55<br>72	119	55	123	59<br>76	formic acid
his	155	156	110<br>83	159	113	162	115<br>NA	formic acid
ser	105	106	60	107	61	109	62	formic acid
phe	165	166	120<br>103	167	103	175	128<br>111	formic acid
tyr	181	182	136<br>91	183	137	191	144<br>98	formic acid
trp	204	205	188<br>146	207	147	216	199<br>155	formic acid
ile	131	132	69	133	69	138	74	formic acid
leu/ile[§]	131	132	86	133	87	138	91	formic acid
thr/hs	119	120	74<br>56	121	75	124	77<br>59	formic acid
asn	132	133	74<br>87	NA	NA	NA	NA	ammonium acetate
		131	114	133	115	135	118	tributylamine
asp	133	132	88<br>115	133	89	136	91<br>119	tributylamine
gln	146	145	127<br>109	147	129	150	132	tributylamine
glu	147	146	102<br>128	147	129	151	133	tributylamine
FBP	340	339	97			345	97	tributylamine
2/3-PG	186	185	97			188	97	tributylamine
pyruvate	88	87	43			90	45	tributylamine
citrate	192	191	87			197	90	tributylamine
α-KG	146	145	101			150	105	tributylamine
succinate	118	117	73			121	76	tributylamine
fumarate	116	115	71			119	74	tributylamine
malate	134	133	115			137	119	tributylamine

[§] Leu and ile can be distinguished by their retention time.

NA: not available.

2/3-PG: 2/3- phosphoglycerate

FBP: fructose-bisphosphate

α-KG: alpha-ketoglutarate

Supplementary Table 3. MRM transitions for AB SCIEX 6500⁺

Metabolites	Parental mass	[U-^{12}C] Q_1	[U-^{12}C] Q_3	[U-^{13}C] Q_1	[U-^{13}C] Q_3
phe	165	166	120<br>103	175	111
tyr	181	182	165<br>136	191	144
trp	204	205	188<br>146	216	155
asp	133	134	88<br>74	138	91<br>76
gln	146	147	130<br>84	152	135<br>88
glu	147	148	130<br>84	153	135<br>88
val	117	118	55	123	59
leu/ile§	131	132	86	138	91
pro	115	116	70	121	74
met	149	150	133	155	138
arg	174	175	70	181	74
his	155	156	110	162	115
ser	105	106	60	109	62
thr	119	120	74	124	77
asn	132	133	74	137	76

§ Leu and ile can be distinguished by their retention time.

Supplementary references

1 van Dijken, J. P. *et al.* An interlaboratory comparison of physiological and genetic properties of four *Saccharomyces cerevisiae* strains. *Enzyme Microb. Technol.* **26**, 706-714 (2000).

2 Klionsky, D. J. Monitoring autophagy in yeast: the Pho8Delta60 assay. *Methods Mol. Biol.* **390**, 363-371 (2007).

3 Longtine, M. S. *et al.* Additional modules for versatile and economical PCR-based gene deletion and modification in *Saccharomyces cerevisiae*. *Yeast* **14**, 953-961, doi:10.1002/(SICI)1097-0061(199807)14:10<953::AID-YEA293>3.0.CO;2-U (1998).

4 Gueldener, U., Heinisch, J., Koehler, G. J., Voss, D. & Hegemann, J. H. A second set of loxP marker cassettes for Cre-mediated multiple gene knockouts in budding yeast. *Nucleic Acids Res.* **30**, e23, doi:10.1093/nar/30.6.e23 (2002).
