# Supplementary material for: Autophagy sustains glutamate and aspartate synthesis in Saccharomyces cerevisiae during nitrogen starvation
Source: Nat Commun. 2021 Jan 4;12:57. doi: 10.1038/s41467-020-20253-6 (PMC7782722; doi:10.1038/s41467-020-20253-6)
Supplement: Supplementary file 3 — Reporting Summary [file 41467_2020_20253_MOESM3_ESM.pdf]

nature research

Corresponding author(s): Benjamin P. Tu

Last updated by author(s): Nov 18, 2020

Reporting Summary

Nature Research wishes to improve the reproducibility of the work that we publish. This form provides structure for consistency and transparency in reporting. For further information on Nature Research policies, see our Editorial Policies and the Editorial Policy Checklist.

Statistics

For all statistical analyses, confirm that the following items are present in the figure legend, table legend, main text, or Methods section.

n/a	Confirmed	
☐	☒	The exact sample size (n) for each experimental group/condition, given as a discrete number and unit of measurement
☐	☒	A statement on whether measurements were taken from distinct samples or whether the same sample was measured repeatedly
☐	☒	The statistical test(s) used AND whether they are one- or two-sided<br>*Only common tests should be described solely by name; describe more complex techniques in the Methods section.*
☒	☐	A description of all covariates tested
☐	☒	A description of any assumptions or corrections, such as tests of normality and adjustment for multiple comparisons
☐	☒	A full description of the statistical parameters including central tendency (e.g. means) or other basic estimates (e.g. regression coefficient) AND variation (e.g. standard deviation) or associated estimates of uncertainty (e.g. confidence intervals)
☐	☒	For null hypothesis testing, the test statistic (e.g. F, t, r) with confidence intervals, effect sizes, degrees of freedom and P value noted<br>*Give P values as exact values whenever suitable.*
☒	☐	For Bayesian analysis, information on the choice of priors and Markov chain Monte Carlo settings
☒	☐	For hierarchical and complex designs, identification of the appropriate level for tests and full reporting of outcomes
☒	☐	Estimates of effect sizes (e.g. Cohen's d, Pearson's r), indicating how they were calculated

Our web collection on statistics for biologists contains articles on many of the points above.

Software and code

Policy information about availability of computer code

Data collection	Metabolites were quantified using the AB SCIEX Analyst software (version 1.6.2 and 1.6.3) by calculating total peak areas.
Data analysis	N.A.

For manuscripts utilizing custom algorithms or software that are central to the research but not yet described in published literature, software must be made available to editors and reviewers. We strongly encourage code deposition in a community repository (e.g. GitHub). See the Nature Research guidelines for submitting code & software for further information.

Data

Policy information about availability of data

All manuscripts must include a data availability statement. This statement should provide the following information, where applicable:

- Accession codes, unique identifiers, or web links for publicly available datasets
- A list of figures that have associated raw data
- A description of any restrictions on data availability

The data that support the findings of this study are within the article and Supplementary Figures. The source data for Figures 1b, 2b, 3a, 3b, 4a-4c, 5c-5e, 5g, 6b-6d, 7c-7j, 8b-8i, and Supplementary Figures 1a-1d, 2b, 3a-3c, 4a, 4b, 5a-5c, 6b, 6c, 7b-7d, 8, 9b, 10b-10e, 11, 12a, 12b, 13b, 14b-14f, 15b-15g, 16b, 16c, 17c-17g are provided as a Source Data file. All other data and protocols are available from the corresponding author upon reasonable request. Source data are provided with this manuscript.

Field-specific reporting

Please select the one below that is the best fit for your research. If you are not sure, read the appropriate sections before making your selection.

☒ Life sciences ☐ Behavioural & social sciences ☐ Ecological, evolutionary & environmental sciences

For a reference copy of the document with all sections, see nature.com/documents/nr-reporting-summary-flat.pdf

Life sciences study design

All studies must disclose on these points even when the disclosure is negative.

Sample size	No sample-size calculation was performed. Typically two-three independent replicates were analyzed and sometimes more replicates were included, because these numbers were typically sufficient to give statistical power.
Data exclusions	No data were excluded from analysis.
Replication	All the experiments were performed independently, unless otherwise stated (Extended Fig. 5b). Therefore, all data (except for those reported in Extended Fig. 5b) were acquired from biological replicates. The core experiments of this work have been repeated numerous times across a period of three years and all replicates were consistent.
Randomization	No deliberate randomization was performed, because it was not relevant. We were constantly examining tens of millions of yeast cells for each genotype in each experiment, which did not allow for randomization. However, we did try to use different mutant clones and to randomize sample vials when measuring metabolites using LC-MS/MS.
Blinding	No blinding was performed, because the differences between WT and mutants were obvious. With or without blinding, one could report these differences without any biases.

Reporting for specific materials, systems and methods

We require information from authors about some types of materials, experimental systems and methods used in many studies. Here, indicate whether each material, system or method listed is relevant to your study. If you are not sure if a list item applies to your research, read the appropriate section before selecting a response.

Materials & experimental systems

n/a	Involved in the study	
☐	☒	Antibodies
☒	☐	Eukaryotic cell lines
☒	☐	Palaeontology and archaeology
☒	☐	Animals and other organisms
☒	☐	Human research participants
☒	☐	Clinical data
☒	☐	Dual use research of concern

Methods

n/a	Involved in the study	
☒	☐	ChIP-seq
☒	☐	Flow cytometry
☒	☐	MRI-based neuroimaging

Antibodies

Antibodies used	1. Mouse monoclonal α-FLAG (clone M2) from Sigma, cat. #: F1804 (https://www.sigmaaldrich.com/catalog/product/sigma/f1804?lang=en&region=US). Lot #: SLCD3524. It was used at 1:3,000 dilution.<br><br>2. Rabbit α-FLAG antibody from Cell Signaling, cat. #2368S. (https://www.cellsignal.com/products/primary-antibodies/dykddddk-tag-antibody-binds-to-same-epitope-as-sigma-s-anti-flag-m2-antibody/2368). Lot # 12. It was used at 1:3,000 dilution.<br><br>3. Rabbit polyclonal α-Rpn10p from Abcam, cat. #: ab98843 (https://www.abcam.com/rpn10sun1-antibody-ab98843.html?productWallTab=ShowAll). Lot #: GR96957. It was used at 1:40,000 dilution.
Validation	1. The mouse monoclonal α-FLAG antibody: from the listed Sigma website: For highly sensitive and specific detection of FLAG fusion proteins by immunoblotting, immunoprecipitation (IP), immunohistochemistry, immunofluorescence and immunocytochemistry. Optimized for single banded detection of FLAG fusion proteins in mammalian, plant, and bacterial expression systems. We have also verified that it can react with FLAG-tagged proteins, as we could detect WB signals at the expected molecular weights (Supplementary Figure 10e). NOTE: α-FLAG antibody is species independent, because it recognizes the FLAG epitope (DYKDDDDK), which was introduced to the C-terminus of a protein of interest in our work.<br><br>2. The rabbit α-FLAG antibody: from the listed Cell Signaling website: DYKDDDDK Tag Antibody detects exogenously expressed DYKDDDDK proteins in cells. The antibody recognizes the DYKDDDDK peptide (the same epitope recognized by Sigma's Anti-FLAG® antibodies) fused to either the amino- or carboxy-terminus of targeted proteins. The binding specificity of this antibody is NOT

dependent on the presence of divalent metal cations. We have also verified that it can react with FLAG-tagged proteins, as we could detect WB signals at the expected molecular weights (Supplementary Figure 10e). NOTE: α-FLAG antibody is species independent, because it recognizes the FLAG epitope (DYKDDDDK), which was introduced to the C-terminus of a protein of interest in our work.

3. α-Rpn10p: from the listed Abcam website: Tested applications: Suitable for: WB; Species reactivity: Reacts with: Saccharomyces cerevisiae. We have also verified that it can react with the Rpn10p, as we could detect WB signals at the expected molecular weight (Supplementary Figure 10e).
